# Development of a web-based calculator to predict three-month mortality among patients with bone metastases from cancer of unknown primary: An internally and externally validated study using machine-learning techniques

**DOI:** 10.3389/fonc.2022.1095059

**Published:** 2022-12-07

**Authors:** Yunpeng Cui, Qiwei Wang, Xuedong Shi, Qianwen Ye, Mingxing Lei, Bailin Wang

**Affiliations:** ^1^ Department of Orthopedic Surgery, Peking University First Hospital, Beijing, China; ^2^ Department of Oncology, Hainan Hospital of PLA General Hospital, Sanya, China; ^3^ Department of Orthopedic Surgery, Hainan Hospital of PLA General Hospital, Sanya, China; ^4^ Chinese PLA Medical School, Beijing, China; ^5^ Department of Thoracic Surgery, Hainan Hospital of PLA General Hospital, Sanya, China

**Keywords:** bone metastasis, cancer of unknown primary, survival estimation, machine learning, risk stratification

## Abstract

**Background:**

Individualized therapeutic strategies can be carried out under the guidance of expected lifespan, hence survival prediction is important. Nonetheless, reliable survival estimation in individuals with bone metastases from cancer of unknown primary (CUP) is still scarce. The objective of the study is to construct a model as well as a web-based calculator to predict three-month mortality among bone metastasis patients with CUP using machine learning-based techniques.

**Methods:**

This study enrolled 1010 patients from a large oncological database, the Surveillance, Epidemiology, and End Results (SEER) database, in the United States between 2010 and 2018. The entire patient population was classified into two cohorts at random: a training cohort (n=600, 60%) and a validation cohort (410, 40%). Patients from the validation cohort were used to validate models after they had been developed using the four machine learning approaches of random forest, gradient boosting machine, decision tree, and eXGBoosting machine on patients from the training cohort. In addition, 101 patients from two large teaching hospital were served as an external validation cohort. To evaluate each model’s ability to predict the outcome, prediction measures such as area under the receiver operating characteristic (AUROC) curves, accuracy, and Youden index were generated. The study’s risk stratification was done using the best cut-off value. The Streamlit software was used to establish a web-based calculator.

**Results:**

The three-month mortality was 72.38% (731/1010) in the entire cohort. The multivariate analysis revealed that older age (P=0.031), lung metastasis (P=0.012), and liver metastasis (P=0.008) were risk contributors for three-month mortality, while radiation (P=0.002) and chemotherapy (P<0.001) were protective factors. The random forest model showed the highest area under curve (AUC) value (0.796, 95% CI: 0.746-0.847), the second-highest precision (0.876) and accuracy (0.778), and the highest Youden index (1.486), in comparison to the other three machine learning approaches. The AUC value was 0.748 (95% CI: 0.653-0.843) and the accuracy was 0.745, according to the external validation cohort. Based on the random forest model, a web calculator was established: https://starxueshu-codeok-main-8jv2ws.streamlitapp.com/. When compared to patients in the low-risk groups, patients in the high-risk groups had a 1.99 times higher chance of dying within three months in the internal validation cohort and a 2.37 times higher chance in the external validation cohort (Both P<0.001).

**Conclusions:**

The random forest model has promising performance with favorable discrimination and calibration. This study suggests a web-based calculator based on the random forest model to estimate the three-month mortality among bone metastases from CUP, and it may be a helpful tool to direct clinical decision-making, inform patients about their prognosis, and facilitate therapeutic communication between patients and physicians.

## Introduction

Cancers of unknown primary (CUP) are metastatic malignancies with verified histology, whereas routine assessments and imaging techniques are unable to identify the primary cancer site (1). According to estimates, CUP occurs in 1% to 5% of all malignant neoplasms (2, 3), hence it is not exceptionally rare. CUP is featured by early and aggressive metastasis (4), such as bone metastases, and up to 30% bone metastases had unknown origin at the time of diagnosis although thorough physical examinations, laboratory tests, and contemporary radiological images were conducted (5).

CUP is still a cancer group of very dismal outcome (6), and CUP patient’s prognoses regrettably obtained minimal improvement for recent decades, despite the emergence of precision oncology which is able to identify the putative origin of the CUP (6). Currently, the appropriate treatments for CUP required evaluation of prognosis. In general, patients with a favorable outcome (20% of CUP patients) were advised to receive locoregional treatment or systemic chemotherapy, while those with an unfavorable outcome (80% of CUP patients) were treated with empirical chemotherapy, and locoregional therapy such as surgical remove of bone metastasis should not be performed in this circumstance (2). Thus, estimating the prognosis is crucial for those patients.

To elaborate, accurate and individualized prediction of survival is vital to making clinical decision for the treatment of CUP patients with bone metastases. Surgery shouldn’t be performed on patients who have a low chance of survival since it could cause more harm than good. In particular, palliative therapies should be used to treat patients with a survival time of fewer than three months (7–9). It should be noted that early appropriate prognostic discussions with patients could lead to better medical education of therapeutic goals and life expectancy (10). Although a multitude of studies proposed individual prognostic scoring systems to predict survival prognosis, survival classification models among CUP patients with bone metastases were really limited (11). Besides, oncologists did not frequently encounter CUP patients, which restricted physician’s experience and intuitions that they could depend on for prognostic discussion.

Therefore, this study attempted to propose and validate an accurate model to predict three-month mortality among CUP patients with bone metastases. Because of individualized prognostic evaluation and favorable predictive performance, machine learning approaches were widely used among oncologists to predict the survival prognosis of cancer patients (12, 13). Additionally, to encourage clinical implementation, the model will be presented as the format of a web-based calculator that is user-friendly for doctors to use. The study’s hypothesis was that the model could be developed after choosing relevant risk factors as model parameters, utilizing machine learning to achieve the excellent accuracy of models, and establishing web calculator to increase utility.

## Patients and methods

### Patient selection

**Figure 1:**
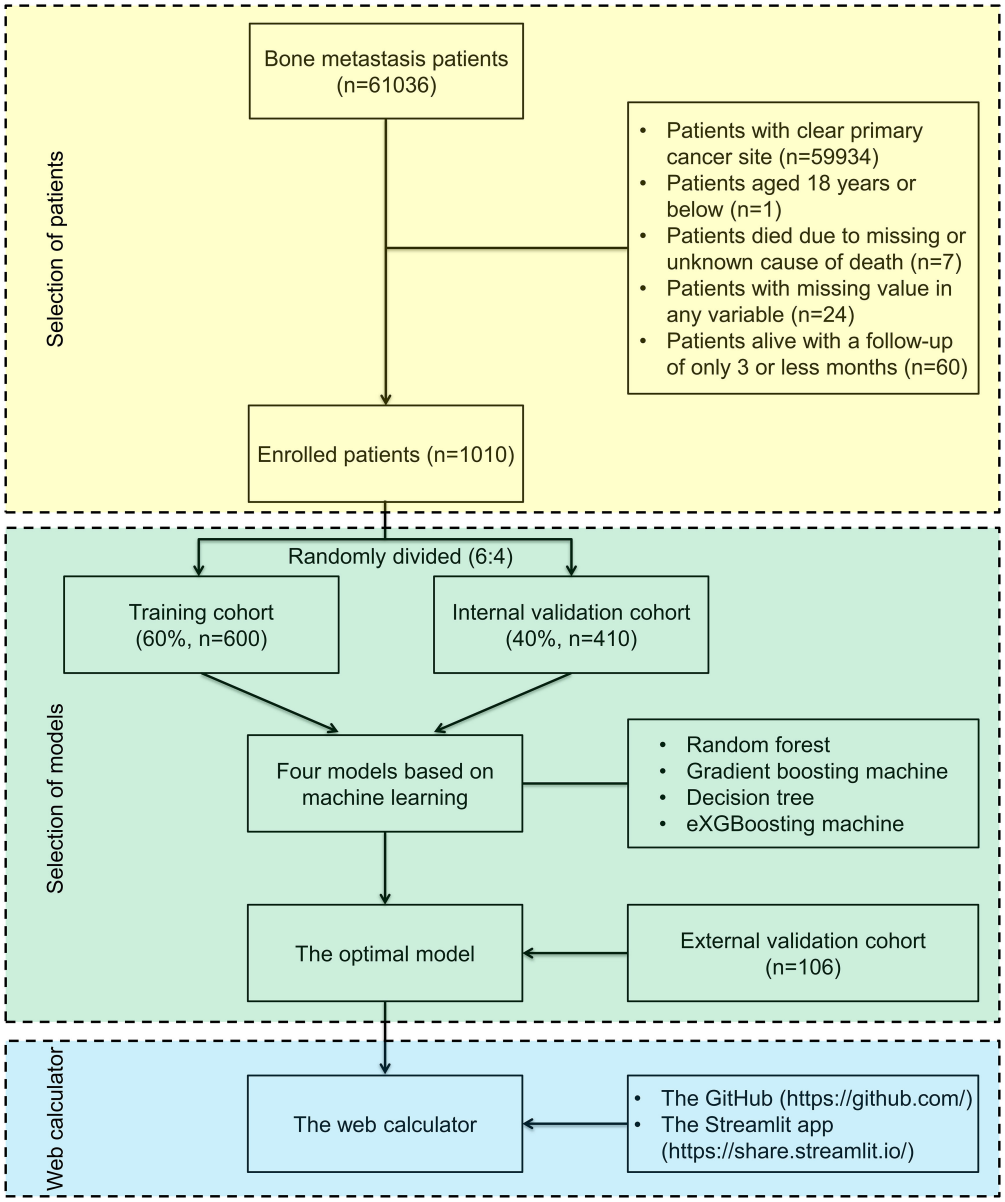
Flow diagram outlining patients and methods. The yellow area indicates selection of patients, the green area indicates selection of the optimal machine-learning model in the study, and the blue area indicates establishment of a web-based calculator.

In the Surveillance, Epidemiology and End Results (SEER) database, 61036 individuals with bone metastases between 2010 and 2018 were examined for this investigation. The SEER database is a project of the National Cancer Institute and, in an effort to lessen the burden of cancer, it provides an authoritative source of information on cancer incidence and survival rate of 28.0% of the population in the United States. The SEER program is supported by the Surveillance Research Program in NCI’s Division of Cancer Control and Population Sciences (http://seer.cancer.gov). SEER gathers data on cancer cases from a variety of locations and sources in the whole United States. The SEER*Stat Version 8.4.0.1 program was used in this investigation to retrieve information about individuals with bone metastases from the SEER database (2010–2018). CUP Patients were included in analysis. Patients were disqualified if they met the following criteria (1): 18 years old or less (2), death due to missing or unknown cause (3), having missing values in needed variable, and (4) having a follow-up of 3 months or less (**Figure 1**). Based on the above inclusive and exclusive criteria, 1010 patients were enrolled, and a 6:4 split of the entire patient population was randomly assigned to a training group (n=600) and a validation cohort (n=410). Patients from the validation cohort were used to validate the model, which had been developed with patients from the training cohort. A series of 106 CUP patients with bone metastases underwent external validation. Between December 2013 and June 2022, these patients were gathered from the Peking University First Hospital and the Hainan hospital of Chinese PLA General Hospital. The study protocol was approved by the Ethics Committee of the Peking University First Hospital and the Hainan hospital of Chinese PLA General Hospital, and written informed consent was waived due to retrospective data in nature.

### Variables and the outcome

The study extracted the following variables: age (years), race (black or other or unknown or white), sex (female or male), brain metastasis (no or unknown or yes), liver metastasis (no or unknown or yes), lung metastasis (no or unknown or yes), radiation (no/unknown or yes), and chemotherapy (no/unknown or yes). Based on the Extent of Disease classification and the American Joint Committee on Cancer, the tumor stage and node stage were recoded. Three-month mortality was defined as patients had a survival outcome of three or less months according to the data in the SEER database.

### Machine-learning models and evaluation of models

This study used four machine learning approaches including random forest (14), gradient boosting machine (15), decision tree (16), and eXGBoosting machine (17) to establish models in the training cohort. Leo Breiman in 2001 introduced random forest, and, using the bagging method (Bootstrap AGGregatING), random forest takes use of a series of decision trees with low reciprocal correlation and randomly selected attributes (14). Gradient boosting machine, introduced by Microsoft, is regarded as an ensemble algorithm, and it is able to provide an efficient use of the gradient boosting algorithm with the primary benefit of dramatic acceleration of the training algorithms (15). Decision tree is a data mining method for classification data, and it is presented as a tree-like structure, and in this structure each internal node indicates a test of a feature and each leaf node indicates a classification (16). eXGBoosting machine is a supervised machine learning model, and it utilizes an improved generalized gradient boosting technique to quickly determine the value of all input features (17).

Prior to modelling, significant variables should be identified by multivariate analysis in the training cohort. In the validation cohort, the nine prediction measures, including area under the receiver operating characteristic (AUROC) curves, discrimination slope, calibration slope, intercept-in-large, sensitivity, specificity, precision, Youden index, and accuracy, were used to evaluate predictive performance of the models. Among the nine metrics, AUROC, calibration, sensitivity, specificity, and accuracy are commonly used to evaluate model’s effectiveness. The optimal model is the one with the most favorable discrimination and calibration.

AUROC was plotted using “pROC” package. Accuracy is better when the area under the curve (AUC) is higher. In details, an AUC value of 0.5 denotes chance, whereas an AUC value of 1.0 denotes complete compliance. Calibration curve was plotted using the “val.prob.ci” function, and the curve was plotted with the predicted probability against actual probability. Decision curve was plotted using the “ggDCA” package, and the curve was used to evaluate the clinical benefit of the model by quantifying the net benefit under different threshold probabilities. In this curve, two reference lines were placed to show the highest clinical cost (treat-for-all plan) and no clinical benefit (treat-for-none plan).

### Establishment of web-based calculator

The optimal machine-learning model was used to construct the web-based calculator. Firstly, the optimal model was saved as the format of PKL document *via* Python software (version 3.9.7). Secondly, the optimal model and corresponding Python code were both uploaded to the GitHub (https://github.com/) website. Lastly, a web calculator can be subsequently deployed after interlinking the Streamlit app (https://share.streamlit.io/) into the GitHub. In the calculator, this study designed a panel of feature selection, an introduction of the web calculator, a submit bottom, and results showing probability of three-month mortality and risk group classification.

### Statistical analysis

All continuous data from the entire study were shown as mean and standard deviation (SD), while all categorical data were shown as proportions. To check for comparability, data from patients in the training and validation cohorts were compared. In the training cohort, a subgroup analysis was done between individuals who experienced three-month mortality and those who did not. Additionally, risk stratification was carried out in the study based on the ideal cut-off value (threshold), and a comparison between low-risk and high-risk groups using Chi-square test. R programming language (version 4.1.2) was used for data visualization and statistical analysis, while Python (version 3.9.7) was used for machine learning operations. A P value of less than 0.05 was regarded as statistically significant, and all P values were two-tailed.

## Results

### Patient’s basic clinical characteristics

A total of 1010 bone metastasis patients were enlisted for analysis based on the inclusive and exclusive criteria. Age was 71.41 years (SD: 13.43 years) on average. The majority patients were white (79.5%) and male (54.3%) (**Table 1**). Only a small fraction of patients suffered from brain metastasis (9.7%), but up to 46.4% of patients had liver metastasis, and 35.0% of patients diagnosed with lung metastasis, indicating that the burden due to metastatic diseases was relatively high. The therapeutic interventions were not so commonly performed since only 26.8% of patients underwent radiation and 20.7% treated with chemotherapy possibly. This could be because the primary origin of cancers was not known, making it difficult to undertake the proper interventions. The median survival time was 1.0 months (95% CI: 0.83-1.17 months) in the entire cohort. In addition, **Table 1** also demonstrated that the baseline characteristics were comparable between the training and validation cohorts (All P>0.05).

**Table 1:** Patient’s demographics and clinical characteristics.

Characteristics	Overall	Cohorts		P-values ^a^
		Training	Validation	
n	1010	600	410	
Age (mean (SD))	71.41 (13.43)	71.17 (14.04)	71.77 (12.50)	0.487
Race (%)				0.619
Black	135 (13.4)	75 (12.5)	60 (14.6)	
White	803 (79.5)	482 (80.3)	321 (78.3)	
Others	72 (7.1)	43 (7.2)	29 (7.1)	
Sex (%)				0.793
Female	462 (45.7)	277 (46.2)	185 (45.1)	
Male	548 (54.3)	323 (53.8)	225 (54.9)	
Brain metastasis (%)				0.553
No	747 (74.0)	440 (73.3)	307 (74.9)	
Unknown	165 (16.3)	104 (17.3)	61 (14.9)	
Yes	98 (9.7)	56 (9.3)	42 (10.2)	
Liver metastasis (%)				0.289
No	431 (42.7)	253 (42.2)	178 (43.4)	
Unknown	110 (10.9)	73 (12.2)	37 (9.0)	
Yes	469 (46.4)	274 (45.7)	195 (47.6)	
Lung metastasis (%)				0.276
No	502 (49.7)	299 (49.8)	203 (49.5)	
Unknown	155 (15.3)	100 (16.7)	55 (13.4)	
Yes	353 (35.0)	201 (33.5)	152 (37.1)	
Radiation (%)				0.277
No/unknown	739 (73.2)	431 (71.8)	308 (75.1)	
Yes	271 (26.8)	169 (28.2)	102 (24.9)	
Chemotherapy (%)				0.832
No/unknown	801 (79.3)	474 (79.0)	327 (79.8)	
Yes	209 (20.7)	126 (21.0)	83 (20.2)	

SD, Standard deviation.

a indicates the continuity adjusted Chi-square test.

### Analyses of three-month mortality

**Figure 2:**
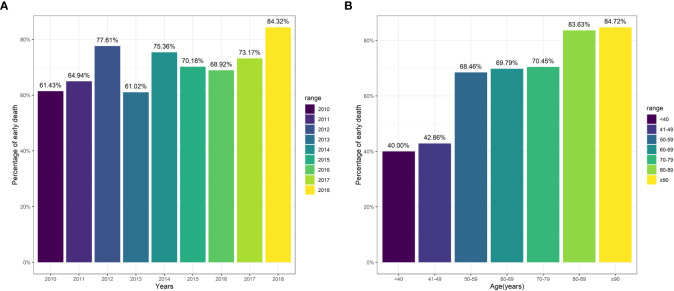
Three-month mortality among patients stratified by variables. **(A)** Year of diagnosis; **(B)** Age.

Of all enrolled patients, up to 72.38% patients passed away at or within three months. Patients with bone metastases from CUP saw relatively steady three-month mortality from 2010 to 2018 (**Figure 2A**). The three-month mortality increased significantly with age (**Figure 2B**). When compared to patients without three-month mortality, patients with three-month mortality had significantly a higher age (73.21 years vs. 66.12 years, P<0.001, **Table 2**) and a higher proportion of white people (82.0% vs. 76.3%, P=0.029). Besides, three-month mortality group had a significantly greater rate of liver metastasis (47.8% vs. 40.5%, P=0.031) and lung metastasis (36.3% vs. 26.6%, P=0.035) and a significant lower rate of receiving radiation (21.1% vs. 45.7%, P<0.001) and chemotherapy (9.4% vs. 49.7%, P<0.001) versus no three-month mortality group.

**Table 2:** Subgroup analysis between patients according to three-month mortality in the training cohort.

Characteristics	Overall	Three-month mortality		P-values ^a^
		No	Yes	
n	600	173	427	
Age (mean (SD))	71.17 (14.04)	66.12 (15.59)	73.21 (12.83)	<0.001
Race (%)				0.029
Black	75 (12.5)	21 (12.1)	54 (12.6)	
White	482 (80.3)	132 (76.3)	350 (82.0)	
Other	43 (7.2)	20 (11.6)	23 (5.4)	
Sex (%)				0.669
Female	277 (46.2)	77 (44.5)	200 (46.8)	
Male	323 (53.8)	96 (55.5)	227 (53.2)	
Brain metastasis (%)				0.785
No	440 (73.3)	127 (73.4)	313 (73.3)	
Unknown	104 (17.3)	28 (16.2)	76 (17.8)	
Yes	56 (9.3)	18 (10.4)	38 (8.9)	
Liver metastasis (%)				0.031
No	253 (42.2)	87 (50.3)	166 (38.9)	
Unknown	73 (12.2)	16 (9.2)	57 (13.3)	
Yes	274 (45.7)	70 (40.5)	204 (47.8)	
Lung metastasis (%)				0.035
No	299 (49.8)	100 (57.8)	199 (46.6)	
Unknown	100 (16.7)	27 (15.6)	73 (17.1)	
Yes	201 (33.5)	46 (26.6)	155 (36.3)	
Radiation (%)				<0.001
No/unknown	431 (71.8)	94 (54.3)	337 (78.9)	
Yes	169 (28.2)	79 (45.7)	90 (21.1)	
Chemotherapy (%)				<0.001
No/unknown	474 (79.0)	87 (50.3)	387 (90.6)	
Yes	126 (21.0)	86 (49.7)	40 (9.4)	


SD, standard deviation.

a indicates the continuity adjusted Chi-square test.

### Selection of features for web calculator

Univariate analysis revealed that age (P<0.001), other race (P=0.044), liver metastasis (P=0.027), lung metastasis (P=0.011), radiation (P<0.001), and chemotherapy (P<0.001) were significantly associated with three-month mortality (**Table 3**). Based on the multivariate analysis, age (P=0.031), liver metastasis (P=0.008), lung metastasis (P=0.012), radiation (P=0.002), and chemotherapy (P<0.001) were significantly relevant to three-month mortality. To be more specific, older age, lung metastasis, and liver metastasis were risk contributors, while radiation and chemotherapy both were protective features. Depending on the multivariate analysis, features for the model was determined. Thus, the four machine learning approaches were used to train and optimize models using the aforementioned five factors.

**Table 3:** Univariate and multivariate analysis of potential risk factors for predicting three-month mortality among bone metastasis from CUP in the training cohort.

Characteristics	Univariate analysis		Multivariate analysis	
	OR (95%CI)	P-value	OR (95%CI)	P-value
n				
Age (mean (SD))	1.04 (1.02-1.05)	<0.001	1.02 (1.00-1.03)	0.031
Race (%)				
Black	Reference		Reference	
White	1.03 (0.60-1.77)	0.912	1.39 (0.73-2.65)	0.311
Other	0.45 (0.20-0.98)	0.044	0.56 (0.23-1.37)	0.204
Sex (%)				
Female	Reference		Reference	
Male	0.91 (0.64-1.30)	0.604	1.06 (0.70-1.61)	0.780
Brain metastasis (%)				
No	Reference		Reference	
Unknown	1.10 (0.68-1.78)	0.693	0.51 (0.22-1.18)	0.117
Yes	0.86 (0.47-1.56)	0.612	1.05 (0.49-2.23)	0.902
Liver metastasis (%)				
No	Reference		Reference	
Unknown	1.87 (1.01-3.44)	0.046	1.85 (0.66-5.20)	0.241
Yes	1.53 (1.05-2.22)	0.027	1.90 (1.18-3.04)	0.008
Lung metastasis (%)				
No	Reference		Reference	
Unknown	1.36 (0.82-2.25)	0.232	1.43 (0.58-3.52)	0.437
Yes	1.69 (1.13-2.54)	0.011	1.93 (1.16-3.22)	0.012
Radiation				
No/unknown (%)	Reference		Reference	
Yes (%)	0.32 (0.22-0.46)	<0.001	0.49 (0.31-0.76)	0.002
Chemotherapy				
No/unknown (%)	Reference		Reference	
Yes (%)	0.10 (0.07-0.16)	<0.001	0.11 (0.07-0.18)	<0.001


OR, odds ratio; CI: confident interval; CUP, cancer of unknown primary; SD, standard deviation.

### Selection of model for web calculator

#### Model construction

The super-parameters were obtained after randomized search with cross validation in each machine learning model, and the full super-parameters were shown in the **Supplementary Table 1**. **Supplementary Figure 1** shows the learning curves of the random forest and gradient boosting machine, and **Supplementary Figure 2** shows the learning curves of the decision tree and eXGBoosting machine. All of these learning curves suggested that underfitting and overfitting were largely avoided after randomized search for appropriate super-parameters.

#### Internal validation

**Figure 3:**
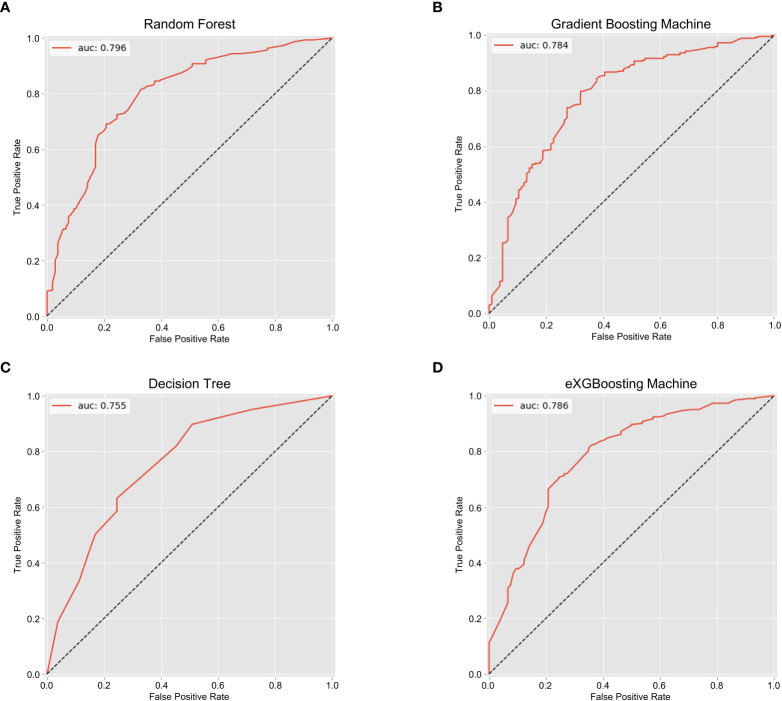
The area under the receiver operating characteristic curves for each approach. **(A)** Random Forest; **(B)** Gradient Boosting Machine; **(C)** Decision Tree; **(D)** eXGBoosting Machine.

**Figure 4:**
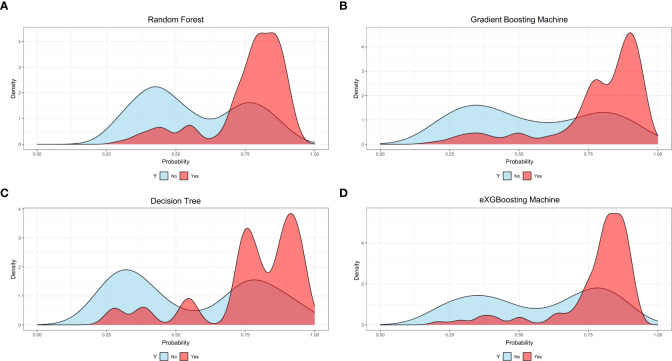
Probability curves for each approach. **(A)** Random Forest; **(B)** Gradient Boosting Machine; **(C)** Decision Tree; **(D)** eXGBoosting Machine.

**Figure 5:**
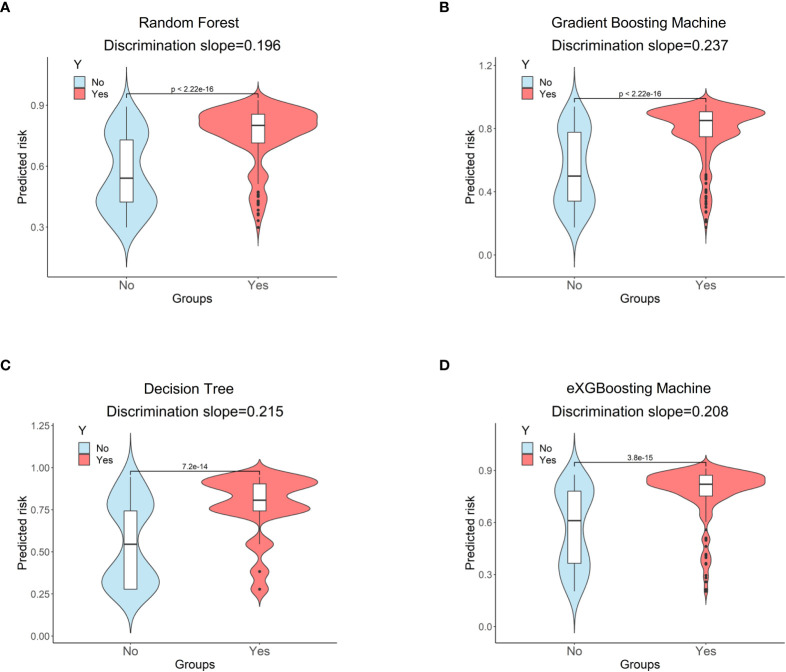
Discrimination slopes for each approach. **(A)** Random Forest; **(B)** Gradient Boosting Machine; **(C)** Decision Tree; **(D)** eXGBoosting Machine.

**Figure 6:**
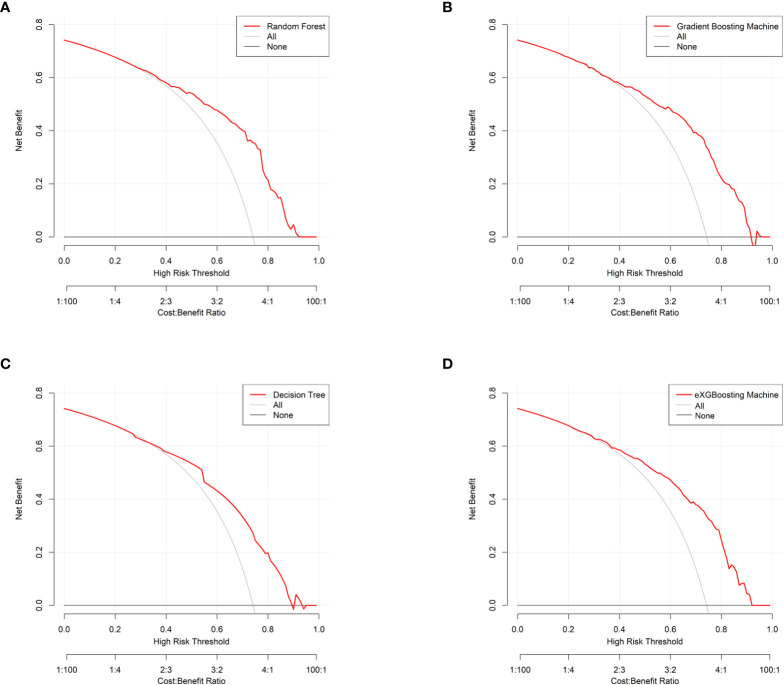
Decision curve analysis for each approach. **(A)** Random Forest; **(B)** Gradient Boosting Machine; **(C)** Decision Tree; **(D)** eXGBoosting Machine.

The area under curve (AUC) values were 0.796 (95% CI: 0.746-0.847, **Figure 3A**) in the random forest model, 0.784 (95% CI: 0.732-0.837, **Figure 3B**) in the gradient boosting machine model, 0.755 (95% CI: 0.701-0.810, **Figure 3C**) in the decision tree model, 0.786 (95% CI: 0.734-0.838, **Figure 3D**) in the eXGBoosting machine model. **Figure 4** shows probability curve of each machine learning model, and it demonstrated that all the four models had favorable discrimination since the two groups were largely separated. The discrimination slope was 0.196 in the random forest (**Figure 5A**), 0.237 in the gradient boosting machine (**Figure 5B**), 0.215 in the decision tree (**Figure 5C**), 0.208 in the eXGBoosting machine approach (**Figure 5D**). The corresponding calibration slopes were 1.36 (**Supplementary Figure 3A**), 0.97 (**Supplementary Figure 3B**), 0.83 (**Supplementary Figure 3C**), and 1.13 (**Supplementary Figure 3D**) respectively. **Figure 6** shows decision curve analysis of all models, and it denoted favorable clinical usefulness of each approach. **Table 4** shows predictive performance of each approach, and it demonstrated that the random forest not only had the highest AUC value, but also the highest Youden index and the second-highest accuracy and precision. Therefore, the random forest model was used as the optimal model in the study. Accordingly, external validation, risk stratification, and the development of a web calculator were conducted using the random forest model.

**Table 4:** Prediction performance of machine learning approaches for predicting three-month mortality among bone metastatic patients from CUP.

Measures	Internal validation cohort				External validation cohort
	Random forest	Gradient boosting machine	Decision tree	eXGBoosting machine	
Intercept-in-large	0.20	0.14	0.18	0.12	-0.95
Calibration slope	1.36	0.97	0.83	1.13	1.25
AUC (95%CI)	0.796 (0.746-0.847)	0.784 (0.732-0.837)	0.755 (0.701-0.810)	0.786 (0.734-0.838)	0.748 (0.653-0.843)
Discrimination slope	0.196	0.237	0.215	0.208	0.113
Specificity	0.670	0.679	0.491	0.642	0.772
Sensitivity	0.816	0.799	0.898	0.822	0.714
Precision	0.876	0.877	0.835	0.868	0.729
Youden	1.486	1.479	1.389	1.464	1.486
Accuracy	0.778	0.768	0.793	0.776	0.745
Threshold	0.711	0.724	0.464	0.723	0.718

AUC, Area under the curve; CI, Confident interval; CUP, cancer of unknown primary; eXGBoosting machine, eXtreme gradient boosting machine.

#### External validation

A series of 106 bone metastasis patients with CUP underwent external validation. The basic clinical characteristics are summarized in **Supplementary Table 2**. The AUC value was 0.748 (95% CI: 0.653-0.843, **Supplementary Figure 4**). The probability curve is depicted in **Supplementary Figure 5**, with discrimination slope being 0.113 (**Supplementary Figure 6**) and the calibration slope being 1.250 (**Supplementary Figure 7**). **Supplementary Figure 8** shows model’s decision curve analysis in the external validation cohort. The above results indicated that the optimal model also exhibited good discrimination and calibration in the external validation cohort.

### Establishment of web calculator

A web calculator was constructed according the optimal model (the random forest model) in the study. Visiting https://starxueshu-codeok-main-8jv2ws.streamlitapp.com/, users is able to access to the online calculator. If the online calculator has gone to sleep (shut down), users are able to access to it *via* clicking “Yes, get this app back up!”. After about 30 seconds, the web-based calculator would be accessible. Users can choose features according to their conditions in the panel of selecting parameters, and then probability of three-month mortality could be obtained by submitting all parameters. In addition, related therapy recommendation was displayed in accordance with the risk stratification. The risk stratification was achieved based on the best cut-off value (71.10%) in the random forest model: Patients in the high-risk group were 1.99 times more likely to suffer from three-month mortality than patients in the low-risk group (P<0.001, **Table 5**) in the internal validation cohort. External validation showed the similar trend (**Table 6**): patients in the high-risk group had 2.37-time odds of three-month mortality than patient in the low-risk group (P<0.001). Additionally, introduction of the model was shown at the end of the interface. **Supplementary Figure 9** shows a screenshot of the web calculator. In the screenshot, a specific example was presented: a 57-year-old patient without liver and lung metastasis did not receive radiation and chemotherapy, and the three-month mortality was up to 72.30%.

**Table 5:** Risk stratification among patients in the internal validation cohort.

Risk groups	Patients (n = 410)	Probability		P-value ^a^
		Predicted	Actual	
Low-risk (≦71.10%)	127	47.25%	44.09% (56/127)	<0.001
High-risk (> 71.10%)	283	81.32%	87.63% (248/283)	

a indicates P-value was obtained from the Chi-square test.

**Table 6:** Risk stratification among patients in the external validation cohort.

Risk groups	Patients (n = 106)	Probability		P-value ^a^
		Predicted	Actual	
Low-risk (≦71.10%)	37	48.43%	24.32% (9/37)	<0.001
High-risk (> 71.10%)	69	76.73%	57.97% (40/69)	

a indicates P-value was obtained from the Chi-square test.

## Discussion

### Main findings

This study found that older age, lung metastasis, and liver metastasis were significant contributors for three-month mortality, with radiation and chemotherapy being protective factors for survival. Furthermore, using machine learning, the study developed an accurate model that was able to predict survival among bone metastases patients from CUP, and its predictive performance showed good discriminative and calibrating ability. The model was incorporated into a web-based calculator to encourage clinical reference and research use. This model might be a useful tool to facilitate personalized survival estimation and do some help to guide clinical decision-making.

### Survival prognosis

In the entire cohort of patients, up to 72.38% patients passed away at or within three months, and this incidence was significantly higher as compared to that among patients with other cancers. It was reported that 33.7% of bone metastasis patients with common cancers suffered from three-month mortality (18) and 44.4% of lung cancer patients with synchronous brain metastasis had early death (a survival outcome of three or less months) (19). Raghav et al. (20) showed that the median survival time of CUP patients was 14.7 months in a retrospective study. Jin et al. (21) found that the median overall survival time of CUP patients was 6.0 months among patients from the SEER database. Chambard et al. (22) reported that bone metastasis patients with lung cancer had a median survival of 7.0 months. Another large retrospective study found that the median survival time of bone metastasis patients with common cancers was 6.0 months (18). As for the cohort of patients in the present study, the median survival time was only 1.0 months. This short survival time could be explained by the evidence that bone metastasis and CUP were all contributors to negatively affecting survival outcome. What’s more, the majority of CUP (80%) had unfavorable prognosis (2). Additionally, this study found that the three-month mortality increased significantly with age, and Shen et al. (19) found the similar trend among lung cancer patients with synchronous brain metastasis.

### Model features

The model features in the study contained five variables: age, lung metastasis, liver metastasis, radiation, and chemotherapy. These variables were also proved to be associated with survival outcome among CUP patients in other studies (20, 23). Furthermore, a recent study showed that gender, Eastern Cooperative Oncology Group performance status, histology, number of metastatic sites, and neutrophil-lymphocyte ratio were independent prognostic factors for survival among CUP patients (20). Huey et al. (24) demonstrated that high neutrophil-to-lymphocyte ratio were associated with worse overall survival among CUP with bone-predominant or lymph node-only disease. Consequently, some measures to improve empirical chemotherapy or radiation, performance status, and neutrophil-lymphocyte ratio would be beneficial to survival prognosis among CUP patients.

### Survival prediction

A number of survival scoring systems had already proposed to predict survival outcome among bone metastasis patients (25), spine metastasis patients (9), and various cancer patients (26–28). As for CUP, in the year of 2021, Raghav et al. (20) developed a model to predict survival prognosis among CUP in a series of 521 patients and validated the model in two cohorts (n=103 and 302). Five independent prognostic factors were included in the model and the model had a C-index of 0.71. In the same year, Jin et al. (21) proposed a model for predicting survival among CUP. A total of 3347 patients were divided into a training cohort and a validation cohort. The C-index of the model was 0.705 in the training cohort and 0.727 in the validation cohort. More recently, in 2022, Yang et al. (29) created a model including six independent prognostic factors (pathology, visceral metastases, Frankel score, weight loss, hemoglobin, and serum tumor markers) to predict survival outcome among spinal metastasis from CUP in a retrospective derivation cohort of 268 patients and this model was validated in a prospective validation cohort of 105 patients. The C-index was 0.775 in the derivation group and 0.771 in the validation cohort. The above prediction models were designed for CUP and might not be applicable in particular bone metastasis patients with CUP. In the present study, the C-index was up to 0.796 based on the random forest model, and the number was the highest as compared to the above studies. Internal and external validation both confirmed that the model had favorable discriminative and calibrating ability.

### Individualized therapeutic strategies

Risk classification of patients was accomplished in the study, and patients could be split into two risk categories based on the ideal threshold, allowing for the personalized execution of therapeutic strategies. Patients in the high-risk categories had a roughly two-fold greater chance than those in the low-risk groups of dying within three months.

For palliative pain relief, patients in the high-risk group may benefit from the best supportive care, short-term radiation, or even minimally invasive procedures like cementoplasty (7). The study’s proposed model, which dose not ask for any additional staff training, can be used clinically to forecast the survival benefit of patients with bone metastases and raise the performance of oncologists and radiologists who aren’t professionals to that of experts.

### Limitations

Although this study was well designed, there were still several drawbacks. To begin with, some variables, such as comorbidity and laboratory data, were not available due to the limitation of the SEER database, and clinically the detailed information on cancer were also unavailable because of the unclear primary cancer site. Incorporating those variables might further improve prediction performance of the model, but the AUC values demonstrated that the model was useful enough to predict three-month mortality. Furthermore, our study aimed at proposing a model with routine clinical data that were widely available and easily accessible. Under such circumstances, it would be more practical for oncologists to apply the model in these situations. In addition, external validation was only performed in a small study, thus the generalization of the model needs further validation. Therefore, although the model was validated and embraced favorable prediction performance, it warrants further extensive revision and validation.

### Conclusions

The random forest model has promising performance with favorable discrimination and calibration. This study suggests a web-based calculator based on the random forest model to estimate the three-month mortality among bone metastases from CUP, and it may be a helpful tool to direct clinical decision-making, inform patients about their prognosis, and facilitate therapeutic communication between patients and physicians. Patients in the high-risk group may better be treated with best supportive care due to very limited survival expectancy.

## Data availability statement

Publicly available datasets were analyzed in this study. This data can be found here: SEER program is supported by the Surveillance Research Program in NCI’s Division of Cancer Control and Population Sciences (http://seer.cancer.gov).

## Ethics statement

The studies involving human participants were reviewed and approved by The study protocol was approved by the Ethics Committee of Peking University First Hospital and Hainan hospital of Chinese PLA General Hospital. Written informed consent for participation was not required for this study in accordance with the national legislation and the institutional requirements.

## Author contributions

All authors conceived and designed the analysis, XS and QW oversaw data collection, YC, QY, and ML performed the analysis and all authors provided clinical interpretation of the findings. XS and ML drafted the manuscript. All authors reviewed, edited and confirmed their acceptance of the final submitted version.

## Supplementary material

Supplementary Figure 1 Learning curves. A. Random Forest; B. Gradient Boosting Machine.

Supplementary Figure 2 Learning curves. C. Decision Tree; D. eXGBoosting Machine.

Supplementary Figure 3 Calibration curves for each approach. **(A)** Random Forest; **(B)** Gradient Boosting Machine; **(C)** Decision Tree; **(D)** eXGBoosting Machine.

Supplementary Figure 4 The area under the receiver operating characteristic curve in the external validation cohort.

Supplementary Figure 5 Probability curve in the external validation cohort.

Supplementary Figure 6 Discrimination slope in the external validation cohort.

Supplementary Figure 7 Calibration curve in the external validation cohort.

Supplementary Figure 8 Decision curve analysis in the external validation cohort.

Supplementary Figure 9 The web calculator.

## References

[1] Lee MS Sanoff HK. Cancer of unknown primary. BMJ (2020) 371:m4050. doi: 10.1136/bmj.m4050 33288500

[2] Pavlidis N Pentheroudakis G. Cancer of unknown primary site. Lancet (2012) 379:1428–35. doi: 10.1016/S0140-6736(11)61178-1 22414598

[3] Rassy E Parent P Lefort F Boussios S Baciarello G Pavlidis N. New rising entities in cancer of unknown primary: Is there a real therapeutic benefit? Crit Rev Oncol Hematol (2020) 147:102882. doi: 10.1016/j.critrevonc.2020.102882 32106012

[4] Rassy E Assi T Pavlidis N. Exploring the biological hallmarks of cancer of unknown primary: where do we stand today? Br J Cancer (2020) 122:1124–32. doi: 10.1038/s41416-019-0723-z PMC7156745 32042068

[5] Piccioli A Maccauro G Spinelli MS Biagini R Rossi B. Bone metastases of unknown origin: epidemiology and principles of management. J Orthop Traumatol (2015) 16:81–6. doi: 10.1007/s10195-015-0344-0 PMC4441638 25726410

[6] Olivier T Fernandez E Labidi-Galy I Dietrich PY Rodriguez-Bravo V Baciarello G. Redefining cancer of unknown primary: Is precision medicine really shifting the paradigm? Cancer Treat Rev (2021) 97:102204. doi: 10.1016/j.ctrv.2021.102204 33866225

[7] Cui YP Shi XD Wang SJ Qin Y Wang BL Che XT. Machine learning approaches for prediction of early death among lung cancer patients with bone metastases using routine clinical characteristics: An analysis of 19,887 patients. Front Public Health (2022) 10:1019168. doi: 10.3389/fpubh.2022.1019168 36276398 PMC9583680

[8] Cui Y Lei M Pan Y Lin Y Shi X. Scoring algorithms for predicting survival prognosis in patients with metastatic spinal disease: The current status and future directions. Clin Spine Surg (2020) 33:296–306. doi: 10.1097/BSD.0000000000001031 32604194

[9] Lei M Li J Liu Y Jiang W Liu S Zhou S. Who are the best candidates for decompressive surgery and spine stabilization in patients with metastatic spinal cord compression? A new scoring system. Spine (Phila Pa 1976) (2016) 41: 1469–76, 41. doi: 10.1097/BRS.0000000000001538 PMC5001136 26937605

[10] Liu PH Landrum MB Weeks JC Huskamp HA Kahn KL He YL. Physicians’ propensity to discuss prognosis is associated with patients' awareness of prognosis for metastatic cancers. J Palliat Med (2014) 17:673–U49. doi: 10.1089/jpm.2013.0460 24742212 PMC4038989

[11] Culine S. Prognostic factors in unknown primary cancer. Semin Oncol (2009) 36:60–4. doi: 10.1053/j.seminoncol.2008.10.004 19179189

[12] Ngiam KY Khor IW. Big data and machine learning algorithms for health-care delivery. Lancet Oncol (2019) 20:e262–73. doi: 10.1016/S1470-2045(19)30149-4 31044724

[13] Tahmassebi A Wengert GJ Helbich TH Bago-Horvath Z Alaei S Bartsch R. Impact of machine learning with multiparametric magnetic resonance imaging of the breast for early prediction of response to neoadjuvant chemotherapy and survival outcomes in breast cancer patients. Invest Radiol (2019) 54:110–7. doi: 10.1097/RLI.0000000000000518 PMC6310100 30358693

[14] Strobl C Malley J Tutz G. An introduction to recursive partitioning: rationale, application, and characteristics of classification and regression trees, bagging, and random forests. Psychol Methods (2009) 14:323–48. doi: 10.1037/a0016973 PMC2927982 19968396

[15] Ke GL Meng Q Finley T Wang TF Chen W Ma WD. Lightgbm: A highly efficient gradient boosting decision tree. in advances in neural information processing systems 30 (NIP 2017) (2017). Available at: https://proceedings.neurips.cc/paper/2017/hash/6449f44a102fde848669bdd9eb6b76fa-Abstract.html (Accessed 22 November 2022).

[16] Che DS Liu Q Rasheed K Tao XP. Decision tree and ensemble learning algorithms with their applications in bioinformatics. Software Tools Algorithms Biol Syst (2011) 696:191–9. doi: 10.1007/978-1-4419-7046-6_19 21431559

[17] Alim M Ye GH Guan P Huang DS Zhou BS Wu W. Comparison of ARIMA model and XGBoost model for prediction of human brucellosis in mainland China: a time-series study. BMJ Open (2020) 10:e039676. doi: 10.1136/bmjopen-2020-039676 PMC7722837 33293308

[18] Phanphaisarn A Patumanond J Settakorn J Chaiyawat P Klangjorhor J Pruksakorn D. Prevalence and survival patterns of patients with bone metastasis from common cancers in Thailand. Asian Pac J Cancer Prev (2016) 17:4335–40. Available at: http://journal.waocp.org/?sid=Entrez:PubMed&id=pmid:27797240&key=2016.17.9.4335. 27797240

[19] Shen H Deng G Chen Q Qian J. The incidence, risk factors and predictive nomograms for early death of lung cancer with synchronous brain metastasis: a retrospective study in the SEER database. BMC Cancer (2021) 21:825. doi: 10.1186/s12885-021-08490-4 34271858 PMC8285786

[20] Raghav K Hwang H Jacome AA Bhang E Willett A Huey RW. Development and validation of a novel nomogram for individualized prediction of survival in cancer of unknown primary. Clin Cancer Res (2021) 27:3414–21. doi: 10.1158/1078-0432.CCR-20-4117 PMC8197749 33858857

[21] Jin YZ Lin MX Luo ZG Hu XC Zhang J. Development and validation of a nomogram for predicting overall survival of patients with cancer of unknown primary: a real-world data analysis. Ann Transl Med (2021) 9:198. doi: 10.21037/atm-20-4826 33708825 PMC7940932

[22] Chambard L Girard N Ollier E Rousseau JC Duboeuf F Carlier MC. Bone, muscle, and metabolic parameters predict survival in patients with synchronous bone metastases from lung cancers. Bone (2018) 108:202–9. doi: 10.1016/j.bone.2018.01.004 29337225

[23] Li X Shao Y Sheng L Zhu J Wang Z Guo K. Risk factors and predictors for tumor site origin in metastatic adenocarcinoma of unknown primary site. Cancer Med (2021) 10:974–88. doi: 10.1002/cam4.3684 PMC7897950 33405390

[24] Huey RW Smaglo BG Estrella JS Matamoros A Overman MJ Varadhachary GR. Cancer of unknown primary presenting as bone-predominant or lymph node-only disease: A clinicopathologic portrait. Oncologist (2021) 26:E650–7. doi: 10.1002/onco.13700 PMC8018327 33524217

[25] Katagiri H Okada R Takagi T Takahashi M Murata H Harada H. New prognostic factors and scoring system for patients with skeletal metastasis. Cancer Med (2014) 3:1359–67. doi: 10.1002/cam4.292 PMC4302686 25044999

[26] Chi C Fan Z Yang B Sun H Zheng Z. The clinical characteristics and prognostic nomogram for head and neck cancer patients with bone metastasis. J Oncol (2021) 2021:5859757. doi: 10.1155/2021/5859757 34616453 PMC8490031

[27] Jia Y Zhang W You S Li M Lei L Chen L. A nomogram for predicting depression in patients with hepatocellular carcinoma: an observational cross-sectional study. Int J Psychiatry Clin Pract (2019) 23:273–80. doi: 10.1080/13651501.2019.1619777 31124729

[28] Wu J Zhang H Li L Hu M Chen L Xu B. A nomogram for predicting overall survival in patients with low-grade endometrial stromal sarcoma: A population-based analysis. Cancer Commun (2020) 40:301–12. doi: 10.1002/cac2.12067 PMC7365459 32558385

[29] Yang M Ma X Wang P Yang J Zhong N Liu Y. Prediction of survival prognosis for spinal metastasis from cancer of unknown primary: Derivation and validation of a nomogram model. Global Spine J (2022) 21925682221103833. doi: 10.1177/21925682221103833 35615968 PMC10676151

