# Supplementary material for: Development of a web-based calculator to predict three-month mortality among patients with bone metastases from cancer of unknown primary: An internally and externally validated study using machine-learning techniques
Source: Front Oncol. 2022 Dec 7;12:1095059. doi: 10.3389/fonc.2022.1095059 (PMC9768185; doi:10.3389/fonc.2022.1095059)
Supplement: Supplementary file 1 [file DataSheet_1.pdf]

Supplementary table 1. Approaches and their Super-parameters.

Approaches	Super-parameters
Random Forest	RandomForestClassifier(max_depth=52, min_samples_leaf=16, min_samples_split=108, n_estimators=8, random_state=42)
Gradient Boosting Machine	GradientBoostingClassifier(max_depth=92, max_features='log2', min_samples_leaf=88, min_samples_split=130, n_estimators=98, random_state=42)
Decision Tree	DecisionTreeClassifier(max_depth=10, max_features='log2', min_samples_leaf=15, min_samples_split=96, random_state=42)
eXGBoosting Machine	XGBClassifier(base_score=0.5, booster='gbtree', colsample_bylevel=1, colsample_bynode=1, colsample_bytree=1, enable_categorical=False, gamma=0, gpu_id=-1, importance_type=None, interaction_constraints='', learning_rate=0.125, max_delta_step=0, max_depth=1, min_child_weight=9, missing=nan, monotone_constraints='()', n_estimators=66, n_jobs=8, num_parallel_tree=1, predictor='auto', random_state=42, reg_alpha=0, reg_lambda=1, scale_pos_weight=1, subsample=1, tree_method='exact', use_label_encoder=False, validate_parameters=1, verbosity=None)

Supplementary table 2. Patient's basic clinical characteristics in the external validation cohort.

Characteristics	Patients (n=106)
Age (Years, mean ± SD)	65.82±13.74
Sex	
Female	34.9% (37/106)
Male	65.1% (69/106)
Lung metastasis	
No	71.7% (76/106)
Unknown	11.3% (12/106)
Yes	17.0% (18/106)
Liver metastasis	
No	67.0% (71/106)
Unknown	9.4% (10/106)
Yes	23.6% (25/106)
Chemotherapy	
No	78.3% (83/106)
Yes	21.7% (23/106)
Radiation	
No	76.4% (81/106)
Yes	23.6% (25/106)
Early death	
No	53.77% (57/106)
Yes	46.23% (49/106)

SD, standard deviation.
