# Supplementary figures and images for: Development of a web-based calculator to predict three-month mortality among patients with bone metastases from cancer of unknown primary: An internally and externally validated study using machine-learning techniques
Source: Front Oncol. 2022 Dec 7;12:1095059. doi: 10.3389/fonc.2022.1095059 (PMC9768185; doi:10.3389/fonc.2022.1095059)

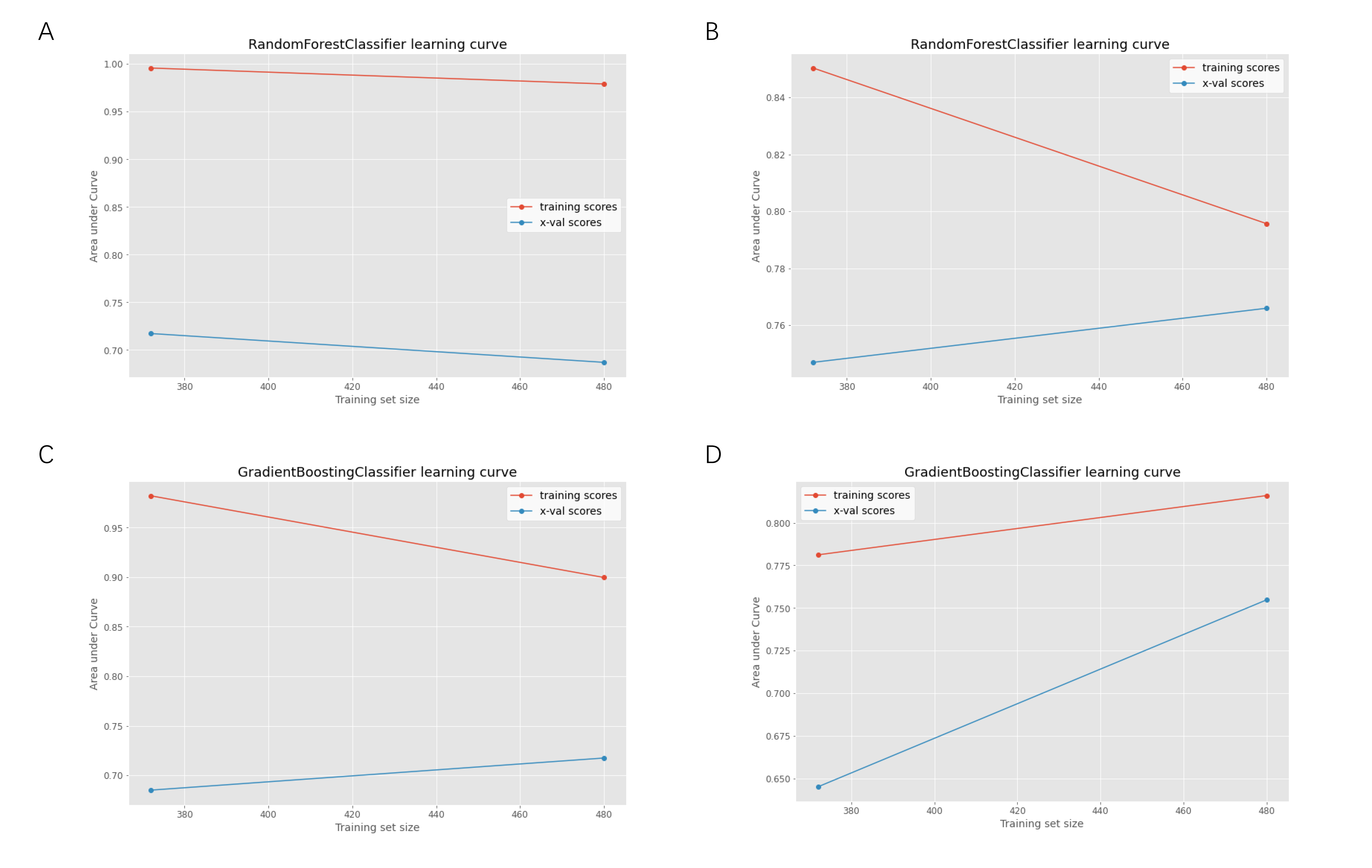

Supplement: Supplementary Figure 1 — Learning curves. A. Random Forest; B. Gradient Boosting Machine. [file Image_1.tif]

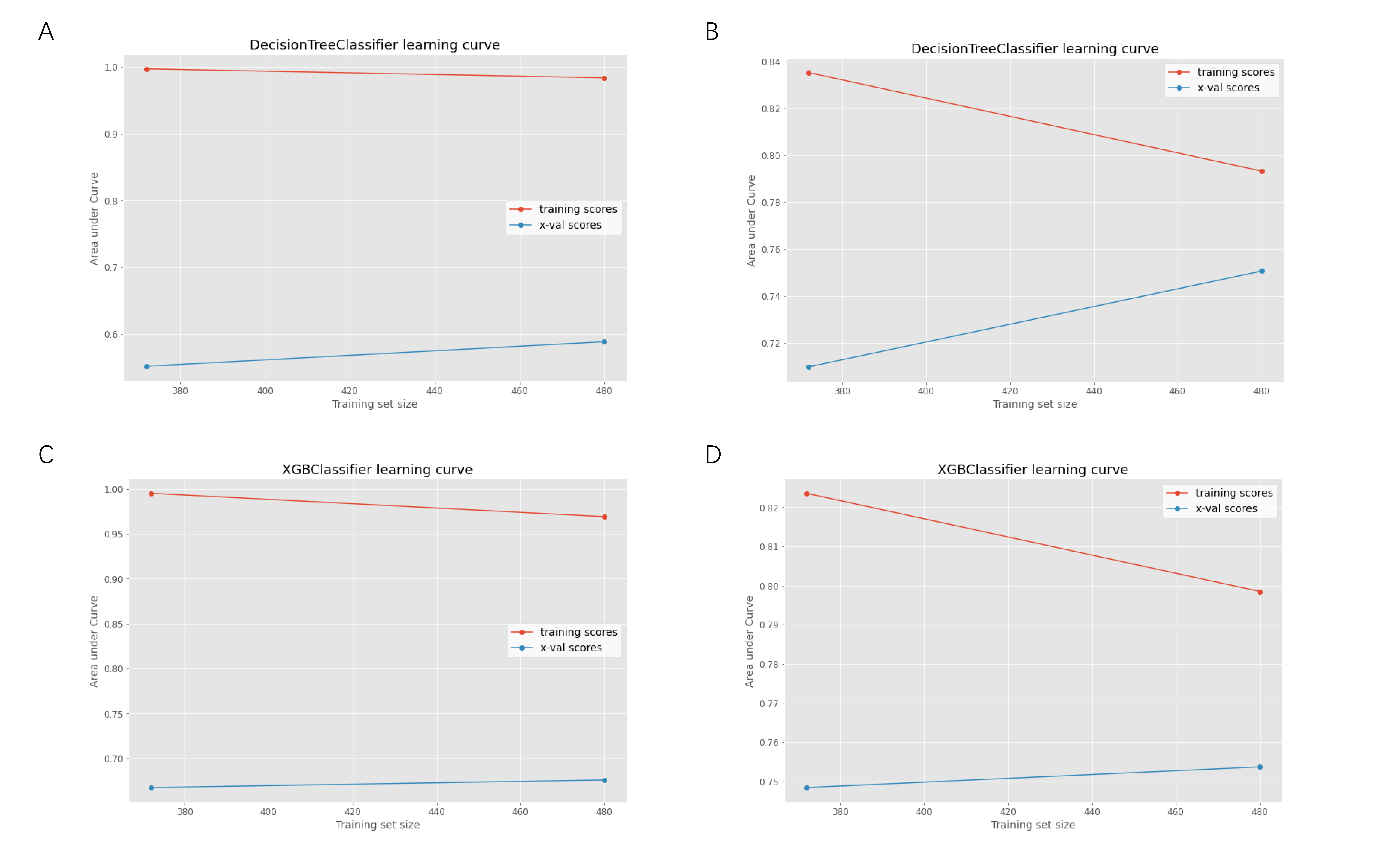

Supplement: Supplementary Figure 2 — Learning curves. C. Decision Tree; D. eXGBoosting Machine. [file Image_2.tif]

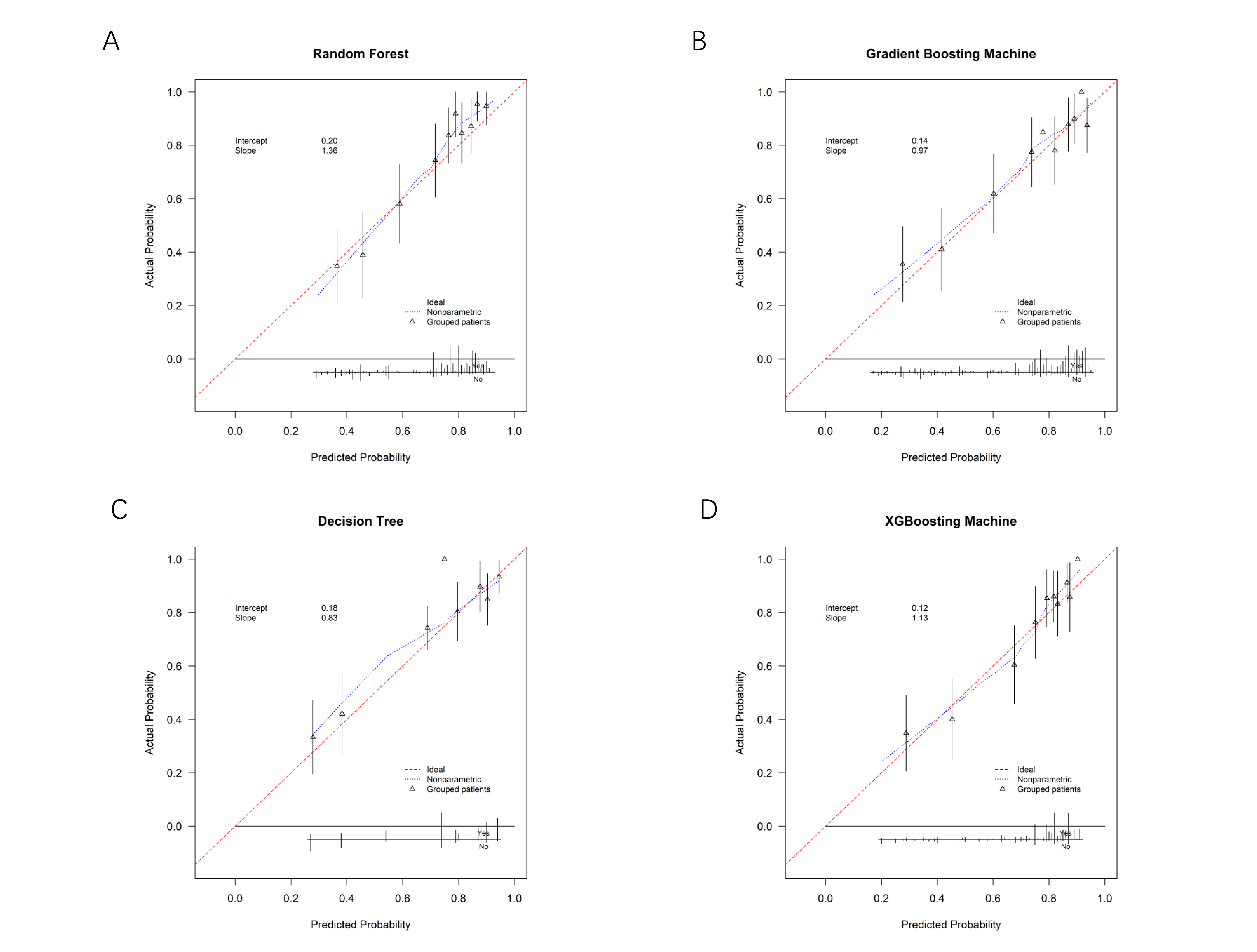

Supplement: Supplementary Figure 3 — Calibration curves for each approach. (A) Random Forest; (B) Gradient Boosting Machine; (C) Decision Tree; (D) eXGBoosting Machine. [file Image_3.tif]

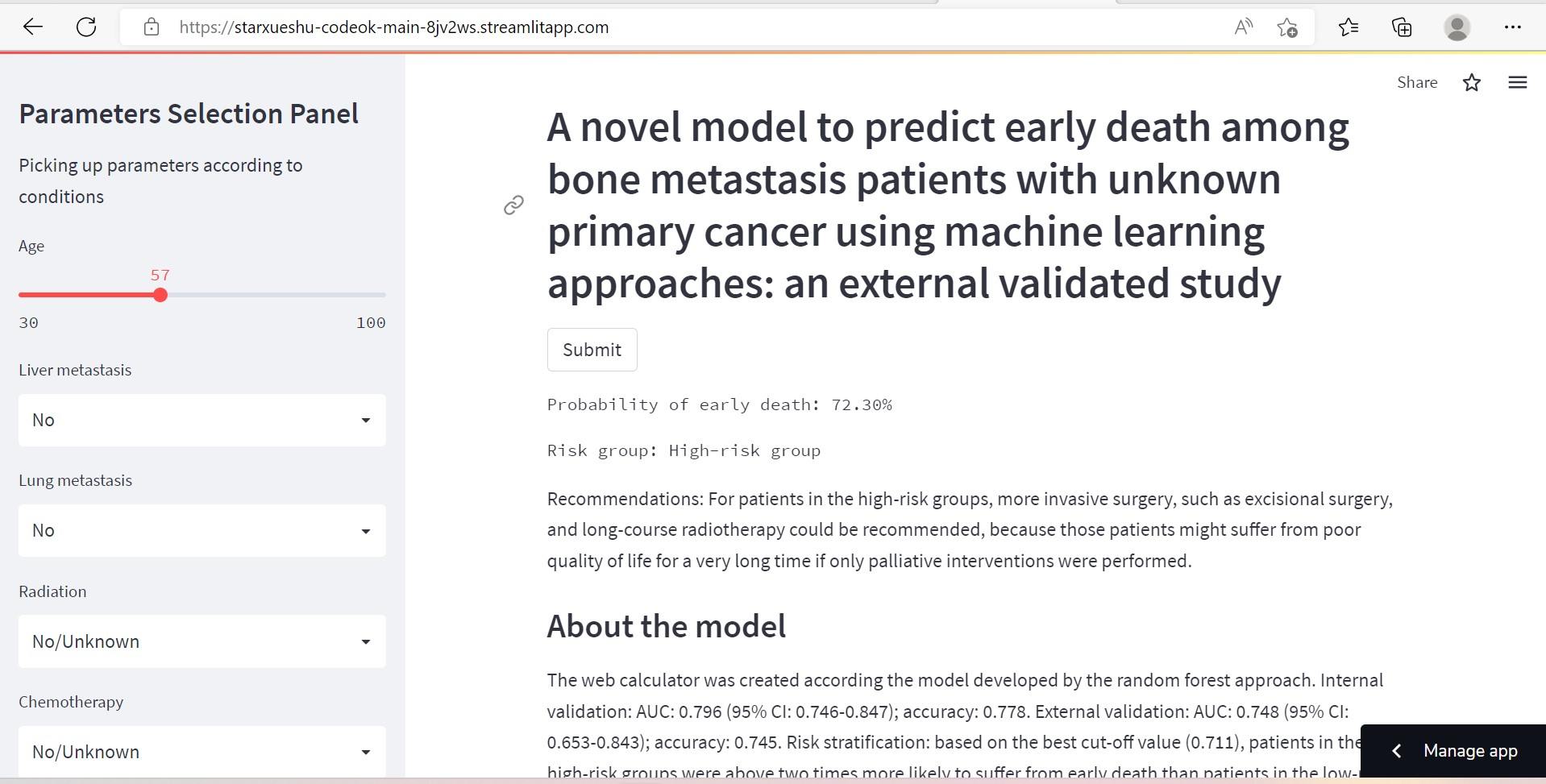

Supplement: Supplementary Figure 9 — The web calculator. [file Image_9.tif]
